# Cytological Features of Inflammatory Mammary Carcinoma in Dogs

**DOI:** 10.3390/vetsci11090389

**Published:** 2024-08-23

**Authors:** Adina-Mihaela Pîrvu, Mario Caniatti, Marta Pieri, Paola Roccabianca, Manuella Militaru

**Affiliations:** 1 Faculty of Veterinary Medicine, University of Agronomic Sciences and Veterinary Medicine of Bucharest, Splaiul Independentei No. 105, 5th District, 050097 Bucharest, Romania; militmanuella@yahoo.com; 2 Department of Veterinary Medicine and Animal Science, Università Degli Studi Di Milano, 26900 Lodi, Italy; mario.caniatti@unimi.it (M.C.); paola.roccabianca@unimi.it (P.R.); 3 Clinica Veterinaria Malpensa, Via Guglielmo Marconi No. 27, 21017 Samarate, Italy; marta.pieri@anicura.it

**Keywords:** inflammatory mammary carcinoma, dog, mammary tumour, cytology, malignancy

## Abstract

**Simple Summary:**

Inflammatory mammary carcinoma (IMC) is the most aggressive and malignant type of mammary tumour, described in humans, dogs, and rarely in cats. This entity may clinically resemble an inflammation but is characterized by a fulminant clinical course, recurrence after surgery, with poor prognosis and survival rates. Cytology is rarely applied for the preliminary diagnosis of mammary tumours, given their complex or mixed morphology in dogs, and especially for IMC, where the diagnosis derives from the association of histopathology and clinical features. Therefore, the aim of this study was to describe cytological features of canine IMC and find possible cytological aspects allowing IMC specific diagnosis and differentiation from other mammary tumour types. Distinctive features of IMC included low cellular cohesiveness, ballooning aspect of some neoplastic cells, marked multinucleation, specific chromatin pattern, and occurrence of squamous metaplasia in some individualised cells or in cells in small groups. Thus, even without clinical information, IMC cytological features allow to suspect and/or to perform a diagnosis of IMC. The study highlights a relevant utility of cytological examination, from which both pathologists and clinicians could benefit, avoiding unnecessary treatments as IMC surgery is not curative.

**Abstract:**

Background: Inflammatory mammary carcinoma (IMC) is the most aggressive and malignant type of mammary carcinoma. As in humans, canine IMC resembles mastitis clinically. However, IMC is highly aggressive with high incidence of metastases and common recurrence after surgery, leading to guarded prognosis and low survival rate. Given the complex morphology of canine mammary tumours, cytological examination is not performed routinely, and IMC diagnosis relies on the association of clinical features and histopathology. The purpose of this study is to describe the characteristics of canine IMC cytology, in an attempt to find possible cytological features that allow differentiation of IMC from other mammary tumour types. Methods: We analysed preoperative cytological samples from 25 dogs with IMC, later confirmed by corroborating clinical and histopathological examinations. Results: Distinct cytological features of canine IMC included scarce cellular cohesiveness, ballooning aspect of neoplastic cells, frequent multinucleation, irregularly dispersed and ropy chromatin pattern, and squamous metaplasia in some individualised cells or those in small groups. Conclusions: Our results indicate that cytological examination can contribute to the diagnosis of IMC and might help differentiate it from other mammary carcinomas, even when clinical data is not available, which is common in cytological routine.

## 1. Introduction

Inflammatory mammary carcinoma (IMC) is one of the most aggressive and malignant mammary carcinoma types and has been described prevalently in humans and dogs [1,2], with very few cases reported in cats [3,4]. IMC is characterized by a rapid clinical course, characterized by high aggressiveness, high tendency to metastasize, and rapid recurrence after surgery, thus having a guarded prognosis and very low survival rates. Relevant clinical features of IMC include cutaneous erythema, swelling, firmness, warmth, and pain of the affected skin and mammary glands, mimicking inflammatory conditions [5].

Interestingly, IMC seems to be more frequent in dogs than in humans, but the true prevalence of IMC is unknown [6]. In dogs, IMC develops almost exclusively in intact females, with no breed predisposition [2], with only one case reported in a male dog [7]. Female dogs with IMC tend to be significantly older compared to dogs with other types of mammary carcinomas [5]. The aetiology of IMC remains unknown, but several predisposing factors have been described, the most relevant being the elevated levels of progesterone during the dioestrus [2].

Prognosis is extremely poor, with an average survival time of 25–35 days for female dogs and no effective treatment available [6,8]. Potential therapeutic efficiency of combinations of COX-2 inhibitor, toceranib, and oral cyclophosphamide has recently been described [9,10]. According to one study [11], IMC seems to have a lower tendency to metastasize to the lungs, liver, kidneys and bones compared to other malignant mammary tumours, and a greater propensity to metastasize to the urinary bladder and other organs of the reproductive tract. Recently, an IMC metastasizing to the bone marrow has been described in a dog [12].

In dogs, two clinical types of IMC have been described: primary IMC, which is the most aggressive form and develops rapidly in patients without previous mammary masses, and secondary IMC, which is the most common; it develops in patients with a history of mammary tumours and has a slightly better evolution compared to primary IMCs [5].

Diagnosis of IMC is highly based on the association of histopathological examination with clinical aspects, as this type of tumour does not represent a histological variant of mammary tumours, or an entity for which the presence of inflammatory cells is typical [2]. Histopathological features include marked pleomorphism and loss of cellular differentiation, phagocytic activity, and invasion of dermal lymphatic vessels by neoplastic cells extensively present at diagnosis, the latter being responsible for the macroscopic inflammatory aspect and considered the pathological hallmark of this entity [2]. Cytological diagnosis of IMC does not always lead to satisfactory results, due to infrequent adequate cellularity or poor sampling [5].

This study aims to evaluate the cytological features of samples obtained from 25 dogs with a later confirmed histologic diagnosis of inflammatory mammary carcinoma. For this reason, we retrospectively analysed cytological specimens, to highlight specific cytological characteristics that could lead to its diagnosis and also differentiation from other mammary tumours.

## 2. Materials and Methods

### 2.1. Case Selection

For this retrospective study, 25 non-consecutive cases of canine IMC were selected. Cases from our archive diagnosed between 1997 and 2002 were used. Only those with a preoperative cytological examination followed by a histological definitive diagnosis were included. Cases with acellular or inconclusive cytological smears and those lacking histopathological confirmation of IMC were excluded. All tumours were routinely processed, paraffin-embedded, and stained with haematoxylin and eosin after surgical excision. The time interval between cytology and histopathology reports was 14–21 days. For each case, data regarding breed, age, and sex were provided.

### 2.2. Cytological Examination

Cytological specimens were obtained by fine-needle aspiration (FNA) or fine-needle capillary (FNC; non-aspiration technique) from mammary neoformations clinically suggestive of inflammatory carcinoma. Slides were air-dried and stained with routine Romanowsky-type stains: Hemacolor^®^ (Merck KGaA, Darmstadt, Germany), Diff-Quick^®^ (Merck KGaA, Darmstadt, Germany), and/or May-Grünwald Giemsa (Merck KGaA, Frankfurt, Germany).

Both cytological and histopathological diagnoses were made at the time the sample was submitted for diagnostic routine. In this study, initial diagnoses were reviewed, and a description of the samples—including assessment of distinctive features—was independently performed by two pathologists (A.M.P., M.C.), as previously described by other authors [13,14,15,16,17]. Due to the retrospective nature of the study and established inclusion criteria, the cytological and histological diagnoses were known by both pathologists. All discrepancies regarding the assessment of the parameters were resolved by consulting a third pathologist (P.R.), and a final agreement was reached. Cellularity of cytologic samples was assessed on low power magnification (10× objective lens) as low (5 or fewer cell clusters), moderate (6–10 cell clusters), and high (more than 10 cell clusters), and ranked on a scale of 1–3, with 1 = low, 2 = moderate, and 3 = good. The presence of inflammatory cells was evaluated by examining the whole slide on low power magnification (10× objective lens), recorded as a percentage, and labelled as low (less than 25% of inflammatory cells), moderate (between 25% and 75% of inflammatory cells), and high (more than 75% of inflammatory cells).

The size of epithelial cells was classified as small (diameter < 2 erythrocytes), medium (diameter 3–4 erythrocytes), and large (diameter > 4 erythrocytes). Other evaluated features included the presence of squamous metaplasia, anisokaryosis, abnormal shapes of the nuclei (other than round and oval), nuclear moulding, multinucleation, abnormal mitotic figures, and increased nuclear-to-cytoplasmic ratio (N/C). For the latter, an N/C > 2/1 was considered high.

Chromatin patterns and irregularities were described and classified according to standard criteria [11], by their assessment on high power magnification (40× and 100× objective lens) in 10 fields. Chromatin irregularities were ranked in scale of 1–3, with 1 = regular, 2 = moderately irregular, and 3 = highly irregular.

Nucleoli were also assessed, based on presence, number, abnormal shapes (other than round and oval), and evidence of macronucleoli (diameter > 4/5 μm). The presence of nucleoli was scored as follows: “−” = not evident, “−/+” = occasionally evident, “+” = evident in all cells. The cohesiveness of neoplastic cells was assessed on low to medium power magnification (10×, 20× objective lens), by examining 10 fields with adequate cellularity and considered low (if more than 50% of the cells are individualised or forming small groups), moderate (if 20–50% are individualised or forming small groups), or high (if less than 20% are individualised or forming groups). Presence of abnormal mitotic figures was quantified based on a previous study on breast cancer [18], counting 10 fields on high power magnification (40× objective lens), resulting in 3 types of results: low (0–1 mitoses), moderate (2–4 mitoses), and high numbers (more than 5 mitoses).

The presence of squamous metaplasia, abnormal nuclear shape, nuclear moulding, multinucleation, increased N/C ratio, abnormal shapes of nucleoli, and macronucleoli were evaluated according to the following: “−” = absent, “+” = mild, “+ +” = moderate, and “+ + +” = marked.

For each case, subsequent histopathological examination confirmed the diagnosis of IMC.

### 2.3. Data Analysis

Descriptive analysis was performed using counts and percentages for categorical variables, and mean and standard deviation (SD) for continuous variables. The Mann–Whitney U test was used to investigate differences in cellularity and cohesiveness depending on the presence of squamous metaplasia.

All statistical analyses were performed using Microsoft Excel (Microsoft Office Professional Plus 2021, Washington, WA, USA). A *p*-value < 0.05 was considered significant.

## 3. Results

### 3.1. Caseload

A total of 25 cases of canine mammary tumours were included in this study. All dogs had clinical presentation highly suggestive of an inflammatory carcinoma that was confirmed by histopathological examination. All lesions were characterized by invasion of dermal lymphatic vessels by neoplastic epithelial cells.

Mean age was 9.92 ± 3.48 (median 11; range, 3.0–16.0 years). Seventeen (68%) dogs were intact, and eight animals (32%) were already neutered at the time of surgery. Studied population comprised eight mixed-breed dogs (32%), seven Dobermans (28%), three German Shepherds (12%), two Boxers (8%), and one French Bulldog (4%), as well as one Cocker Spaniel, one Jack Russell Terrier, one Newfoundland dog, and one Labrador Retriever (4% each).

### 3.2. Cytologycal Smears and Cellular Characteristics

**Figure 1:**
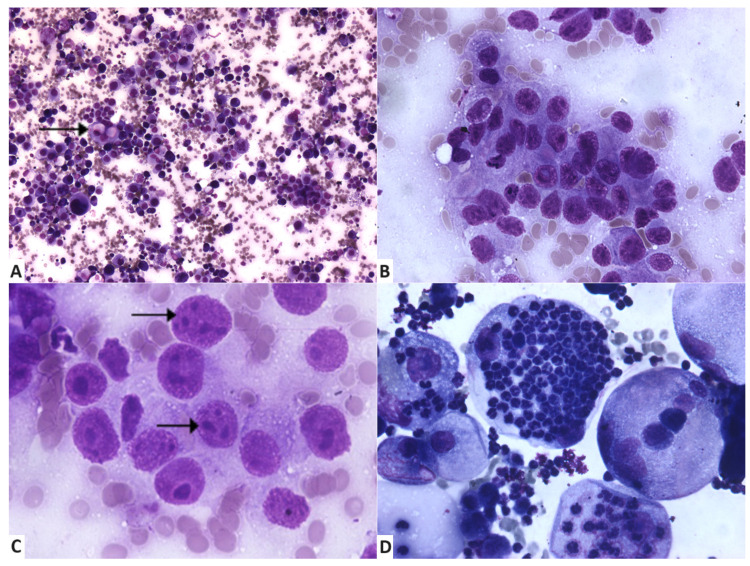
Cytological features of fine-needle aspiration samples from inflammatory mammary carcinoma. (**A**) Highly cellular smear, with epithelial neoplastic cells with marked anisocytosis and anisokaryosis; intracytoplasmic secretory material (arrow) is indicative of secretory origin of malignant tumour. May–Grünwald Giemsa, ×100. (**B**) Group of neoplastic cells with anisokaryosis, multiple evident nucleoli, and a high N/C ratio. May–Grünwald Giemsa, ×400. (**C**) Cells display marked anisonucleolosis, macronucleoli, and ropy chromatin (arrows). May–Grünwald Giemsa, ×400. (**D**) Neoplastic cells with marked atypia and ballooning aspect, associated with the presence of numerous neutrophils. May–Grünwald Giemsa, ×400.

A high cellularity was scored in 64% of the cases (Figure 1A), with the rest having moderate (20%) or low (16%) cellularity.

**Figure 2:**
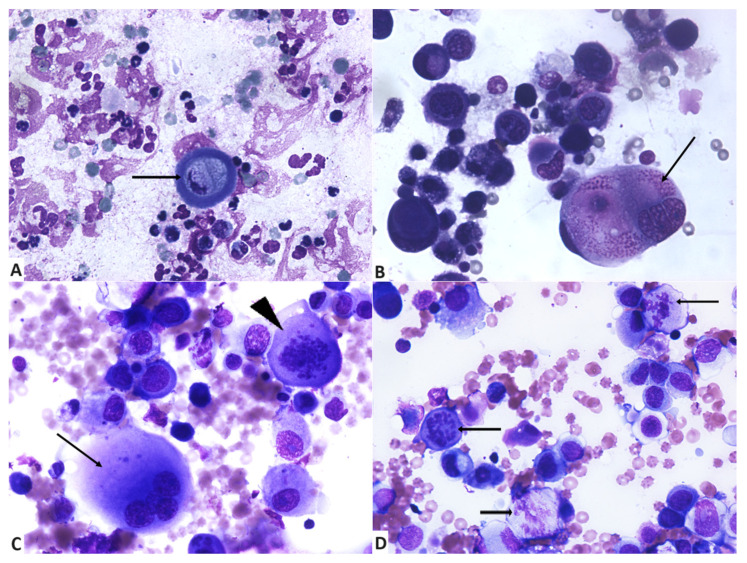
Cytology of inflammatory mammary carcinoma. (**A**) Individualised neoplastic cell with squamous metaplasia (arrow) and numerous viable and degenerated background neutrophils. May–Grünwald Giemsa, ×400. (**B**) Individualised neoplastic cells indicative of loss of cohesiveness, with marked anisokaryosis; giant multinucleated neoplastic cell with intracytoplasmic eosinophilic material and irregular chromatin (arrow). May–Grünwald Giemsa, ×400. (**C**) Low cellular cohesiveness, marked cellular atypia, including a multinucleated neoplastic giant cell (arrow) and one abnormal mitotic figure (arrowhead). May–Grünwald Giemsa, ×400. (**D**) Epithelial malignant tumour cells with atypias and abnormal mitotic figures (arrows). May–Grünwald Giemsa, ×400.

Some samples contained mixed inflammation in addition to the neoplastic epithelial cells. Such inflammatory component was mainly composed of degenerated neutrophils (Figure 1D and Figure 2A) and lesser small mature lymphocytes, plasma cells, and foamy reactive macrophages. Inflammatory cells were present in 13 cases (52%). In six cases, a mild population was observed. Absent or scarce inflammatory cells were recorded in a total of 72% of the cases.

Assessment of cell size revealed that 76% of neoplastic cells were large (n = 19) and 24% (n = 6) were medium sized, with some degree of variation (anisocytosis), which constitutes useful and common criteria of malignancy (Figure 1A and Figure 2B,C). The main cytological features are summarised in Table 1.

Neoplastic cells had low (64%) or moderate (16%) cohesiveness, with the presence of mostly individualised cells and rare groups (Figure 2B–D). Cytological assessment revealed a general lack of glandular, acinar, tubular, or papillary arrangement of cell groups, occasional ballooning aspect of neoplastic cells and also the absence of a spindle-cell component to accompany the epithelial neoplastic cell population. Squamous metaplasia (Figure 2A) was observed in 52% (n = 13) of the cases and scored as mild to absent in 88% of the cases. Cells with squamous metaplasia had either angular borders or were round, with abundant hyalinized, lightly basophilic to aqua-blue cytoplasm, occasionally vacuolized, and with occasionally pyknotic or karyorrhectic nuclei.

Squamous metaplasia was mostly observed in single, individualised cells; our samples contained in total up to 55 single keratinized cells. Low cohesiveness was significantly correlated with the presence of squamous metaplasia (*p* = 0.032). Analyses on cellularity revealed no significant correlation with squamous metaplasia (*p* = 0.163), as shown in Table 2.

Nuclear characteristics of the neoplastic cells in our cases suggest features of malignancy. Anisokaryosis (Figure 1A,B and Figure 2B) was present in all samples. In 56% the cases of anisokaryosis were marked. Abnormal nuclear shapes (Figure 1B,C and Figure 2B) were observed in 96% of cases (n = 24), and assessed as moderate (n = 10), marked (n = 7), and mild (n = 7).

Nuclear moulding was mostly absent (60%), and when present (40%), in only seven of these cases were considered moderate or severe. Multinucleation (Figure 2B,C) was present in 96% of the cases (n = 24) and scored as marked in 12 cases, moderate in 7 cases, and mild in 5. In all samples, neoplastic epithelial cells had a high N/C ratio (>2/1). Ballooning cells and multinucleated neoplastic cells containing large amounts of cytoplasm were excluded when assessing this feature, as they represented a low percentage of neoplastic cells (Figure 1B,D).

In 52% (n = 13) of evaluated cytological smears, abnormal mitotic figures were not observed. When present (n = 12), they were mostly in low numbers (n = 7), followed by high numbers in four cases and moderate numbers in one case (Figure 2C,D).

When examining the chromatin pattern of the neoplastic cells, a preponderance of ropy chromatin (76%) was observed (Figure 1C and Figure 2D). Other patterns were represented by lacy, finely reticular chromatin (12%), finely stippled, smooth chromatin (8%), and coarsely stippled chromatin (4%). Assessing chromatin irregularities (Figure 2B,C), results showed that neoplastic nuclei displayed highly (40%) and moderately (40%) irregularly dispersed chromatin and few had regularly dispersed chromatin (20%). The main nuclear features are summarised in Table 3.

For a better characterization of the neoplastic cells in IMC, nucleolar features were also evaluated. Nucleoli were occasionally evident in 68% of the samples (n = 17); in 16% of the cases they were observed in all the neoplastic cells (n = 4) and were not evident in a very low number of cases (n = 4). Nuclei with fewer than 3 nucleoli were identified in 14 cases; in 3 cases their number was between 3 and 5, and more than 5 in 4 cases. Abnormal nucleolar shapes (Figure 1B,C and Figure 2B) were identified in 64% (n = 16) of the cases, and evaluated as mild in 11 cases, moderate in 3 cases, and marked in only 2 cases. Neoplastic cells with macronucleoli (Figure 1C and Figure 2B) were identified in 72% (n = 18) of the cases, and their presence was marked in eight cases, moderate in three cases, and mild in seven cases. Nucleolar features are listed in Table 4.

## 4. Discussion

The purpose of this study was to describe the cytological characteristics of canine inflammatory mammary carcinoma that could represent specific clues for IMC preliminary diagnosis and enable its differentiation from other mammary tumours.

Diagnostic cytology in veterinary medicine plays an important role in the preliminary information assisting the management of lesions of neoplastic origin [19]. Despite considered relevant in the assessment of mammary lesions in human medicine, mammary cytology in dogs is considered less useful due to the high variability of the results when comparing cytology with the gold standard of histology [14]. Mammary tumours exhibit great heterogeneity in terms of cell types and complex histological architecture, giving cytological examination a limited role in diagnosis establishment [13,14]. Independently from tumour type, well-known disadvantages include inadequate sampling of the tumour site, which contributes to such limitations [13,14]. Also, preoperative cytology is not considered useful by many operators, as the majority of mammary masses are surgically removed without other tumour preoperative investigations [15,20]. These statements are supported by the results of various works on the diagnostic accuracy of cytology in canine mammary tumours in which sensitivity values range from 17% to 88% [21,22,23]. Other studies focusing on the cytological discrimination between benign and malignant mammary tumours in dogs mention a sensitivity ranging from 25% [24] to 95.23% [25] and a specificity from 49% [24] to 96% [26]. These values tend to be even lower when non-diagnostic samples are taken into consideration [27]. The diagnostic accuracy and sensitivity assessed in one study decreased from 96.5% and 96.2% to 63.3% and 60.7%, respectively, when inadequate or suspicious samples were included, with the specificity value not being affected by this change [27]. Only one study on canine IMC includes also a cytological examination [5], and out of 33 cases, only 15 had cytologic evidence of neoplasia.

In the current study, only adequate cytological samples were included. This, along with its retrospective nature and the fact that only cases with later confirmed histological diagnosis of IMC were selected, can be considered the main limitations, as we cannot provide additional data regarding accuracy between cytology and histology in canine mammary tumours, IMC included. It would be interesting in the future to possibly assess the usefulness of cytology in the preliminary diagnosis of canine mammary tumours according to tumour type, as probably simple mammary carcinomas may bear a higher accurate cytological diagnostic rate compared to mixed or complex tumours. This would provide insight on the preliminary diagnosis and cytological diagnostic accuracy of malignant canine simple mammary lesions.

The canine patients included in the present study were all females, with a mean age of 9.9, of which 68% were intact and 32% were neutered. Our results are similar to others described in the literature [5,28], where reported mean ages were 11.4 and 10.5 years, indicating a predisposition of older dogs to develop IMC, as is the case with all types of mammary carcinoma [16,26,29]. However, contrasting results are found in the literature regarding the neutering status, as in one study, the majority (97%) of females with IMC were intact [5] and in another, 70% were spayed [28], confirming the need of more studies on IMC with clinical data included. Still, reviewing data on mammary tumours in dogs, generally, studies indicate intact females as mostly predisposed [14,23,25,26]. The most common breeds in our study included mixed breed (32%), Doberman (28%), and German Shepherd (12%). Our results may suggest a predisposition of this neoplastic entity for Dobermans and German Shepherds, as dogs of mixed breed are fairly common. However, more studies are needed to confirm this statement. In a study of IMC on 33 dogs, those of mixed breed were the most commonly affected, with German Shepherd dogs, Poodles, and Doberman Pinschers among the most common breeds [5]. Other studies [28,29] also mention mixed-breed dogs and German Shepherds as the most common.

Another finding of particular significance in our cases is the presence of mostly individualized neoplastic cells and rare groups. Neoplastic cells had low (64%) or moderate (16%) cohesiveness, a very significant finding from both a diagnostic and pathogenetic point of view. Regarding diagnosis, mammary carcinomas other than inflammatory carcinomas are mostly characterized by the presence of variably-sized groups of neoplastic epithelial cells, while individualised cells or cells in very small groups are not a common finding [2,14,27]. From a pathogenetic point of view, literature mentions inflammatory carcinoma in dogs in the form of various histological types, such as ductal, tubular, or tubulo-papillary [2,3,5,6], with the definitive diagnosis being established on the presence of invasion of the dermal lymphatic vessels, made possible by the loss of cohesion resulting in individualised or very small groups of cells we discovered also in cytology. For that reason, the presence of individualized/small groups of neoplastic cells should be regarded as a pivotal feature in the diagnosis of IMC.

Other cytological findings that could distinguish inflammatory carcinomas from other types of carcinomas were the almost absolute lack of glandular, acinar, or papillary architecture of cells, ballooning aspect of neoplastic cells, and also the absence of a spindle-cell component, which is often found in canine mammary tumours [2,16,22]. The ballooning aspect of neoplastic cells could be interpreted as a probable degenerative phenomenon, on some occasions also associated with a significant number of inflammatory cells, mainly neutrophils. We attributed this feature to the presence of ulceration and/or intratumoural areas of necrosis causing degenerative changes of adjacent neoplastic cells, with karyolysis and intracytoplasmic accumulation of water, resulting in nuclear and cellular swelling [30].

Squamous metaplasia was present in 52% of the examined cases. Despite its slight predominance, we evaluated it as mild to absent in up to 88% of the cases, making it a feature not easily encountered and more likely to go unnoticed without a careful examination of the slide. Interestingly, it was mostly observed in single, individualised cells that are not part of a group, the low cohesiveness being significantly correlated with the squamous metaplasia, as shown by our statistical data. Our samples contained in total up to 55 single keratinized cells, making it, to the authors knowledge, an exceptional finding, with little to absent descriptions in the literature regarding IMC.

Intra or peritumoral inflammatory cells are commonly found in histological samples of canine mammary tumours [15,27]. Data on our cases of IMC revealed that in 72% of the examined samples the inflammatory cells were absent to scarce, their sole presence having been noted in little over half of the cases (52%).

These findings come in contradiction with the literature, where presence of numerous non-degenerated neutrophils and macrophages associated with this neoplasm is mentioned regarding the cytology of IMC [16], leading towards a necessity for more cytological studies focusing on quantifying inflammatory cells in IMC. However, in two studies describing histological aspects of canine IMC, the inflammatory cells were not a prominent feature [2,6] for any of the cases. Despite its name, inflammatory infiltrates are not a distinct feature of this neoplasm, but more the clinical aspect mimicking inflammation of the mammary tissue. Some authors [2] consider that the low number of inflammatory cells in our study could be due to sampling of tumour sites with little to absent inflammatory infiltrates, especially in areas with evidence of central necrosis or ulceration of the skin.

Nuclear characteristics assessed in the present study supported the malignant origin of the tumours and included marked anisokaryosis (56% of the cases) and abnormal nuclear shapes (96% of the cases). Such findings are described in the cytology of inflammatory carcinomas [16]. Our study also focused on nuclear moulding, which we evaluated as mostly absent (60%), and when present (40%), in only seven cases it was considered moderate or severe. This finding is not consistently described in other works on this topic [2,5,14,17,22,23], since this feature is present in many epithelial tumours, including benign epithelial skin tumours [16].

Multinucleation was present in 96% of the cases and considered marked in most of the cases (48%), followed by moderate (28%) and mild (20%). This finding is described as common in inflammatory carcinomas [16], but its quantification has never been made before. The presence of multinucleated cells may also occasionally be seen in all types of mammary carcinomas [11,13,27], but not with the frequency described in our study.

Abnormal mitotic figures were present in 48% of the cases. Our results are slightly different compared to data available in the literature, where they are described as frequent in cases of inflammatory carcinoma [16]. A possible explanation we considered for our study was the sampling of tumour areas that were not most mitotically active.

Currently, the literature lacks data regarding chromatin patterns in IMC to compare our results with. One study focusing on the usefulness of mammary cytology mentions granular and reticular chromatin patterns as being associated with malignant mammary tumours [17]. In our study, the ropy type of chromatin was the most commonly observed (76%) followed by lacy, finely reticular chromatin (12%), finely stippled, smooth chromatin (8%), and coarsely stippled chromatin (4%). Frequently, the chromatin was highly (40%) and moderately (40%) irregularly dispersed. On this matter, we consider as relevant the experience of the clinical pathologist when assessing the chromatin patterns and a reason why future assessment might vary.

When evaluating nucleolar characteristics of IMC, although present in a good percentage of the material studied, the criteria of malignancy were not as high as we expected. Occasionally evident nucleoli made up 68% of the samples and were equally observed in all neoplastic cells or not present in 16%. No other available data on nucleoli in canine IMC seem available, and can be considered a relevant additional morphological clue for future studies on mammary carcinoma malignancy. The number of nucleoli as a prognostic morphological clue in canine mammary tumours is mentioned in one study on histological samples, where a significant difference in the number of nucleoli was found between malignant metastatic and non-metastatic tumours and the benign tumours, with the highest number being present in the malignant metastatic group; they found no differences between the two groups of malignant tumours [31].

Abnormal nucleolar shapes were identified in 64% of the cases and large nuclei in 72%. To the authors’ knowledge, this is the first study on canine IMC focusing on a detailed description of nucleolar features of the neoplastic cells. More studies on such characteristics of canine IMC, both in histology and cytology, are needed to determine other possible distinct features of this tumour and also confirm our findings.

One possible limitation of the present study can be considered the low number of cases, as well as the absence of a follow-up, which, even if not the main focus of the study, as it centres only on cytology, could provide additional details of this tumour. Still, this work is a step forward in the understanding of the cytomorphology of IMC and may contribute to explaining their biological behaviour and help clinicians and pathologists in a better pre-operative identification of this entity.

## Figures and Tables

**Table 1:** Cellular characteristics in cytologic samples.

Examined Criteria	Score	No. of Samples (%)
Cell size	Small	0 (0%)
	Medium	6 (24%)
	Large	19 (76%)
Squamous metaplasia	−	12 (48%)
	+	10 (40%)
	+ +	2 (8%)
	+ + +	1 (4%)
Cellular cohesiveness	Low	16 (64%)
	Moderate	4 (16%)
	High	5 (20%)

Legend: (−) = absent; (+) = mild; (+ +) = moderate; (+ + +) = marked.

**Table 2:** Cytological features in samples without (n = 12) and with (n = 13) squamous metaplasia.

Cytological Features	Samples without Squamous Metaplasia			Samples with Squamous Metaplasia			*p*-Value
Cellularity	Poor	Moderate	High	Poor	Moderate	Good	0.163
	1	2	9	3	3	7	
Cohesiveness	Low	Moderate	High	Low	Moderate	High	0.032 *
	5	3	4	11	1	1	

*p*-value (*) significant at a significance level (α) of 0.05, the Mann–Whitney U test.

**Table 3:** Nuclear characteristics in cytologic samples.

Examined Criteria	Score	No. of Samples (%)
Anisokaryosis	−	0 (0%)
	+	5 (20%)
	+ +	6 (24%)
	+ + +	14 (56%)
Abnormal nuclear shape	−	1 (4%)
	+	7 (28%)
	+ +	10 (40%)
	+ + +	7 (28%)
Nuclear moulding	−	15 (60%)
	+	3 (12%)
	+ +	5 (20%)
	+ + +	2 (8%)
Multinucleation	−	1 (4%)
	+	5 (20%)
	+ +	7 (28%)
	+ + +	12 (48%)
Abnormal mitotic figures	Absent	13 (52%)
	Low numbers	7 (28%)
	Moderate numbers	1 (4%)
	High numbers	4 (16%)

Legend: (−) = absent; (+) = mild; (+ +) = moderate; (+ + +) = marked.

**Table 4:** Nucleolar characteristics in cytologic samples.

Examined Criteria	Score	No. of Samples (%)
Presence of nucleoli	Not evident	4 (16%)
	Occasionally evident	17 (68%)
	Evident	4 (16%)
No. of nucleoli	0	4 (16%)
	<3	14 (64%)
	3–5	3 (12%)
	>5	4 (28%)
Abnormal nucleolar shapes	−	9 (36%)
	+	11 (44%)
	+ +	3 (12%)
	+ + +	2 (8%)
Macronucleoli	−	7 (28%)
	+	7 (28%)
	+ +	3 (12%)
	+ + +	8 (32%)

Legend: (−) = absent; (+) = mild; (+ +) = moderate; (+ + +) = marked.

## Data Availability

The data presented in this study are in the text. The remaining data are available on request from the corresponding author. The data are not publicly available, as they form part of the Ph.D. thesis of the first author, which has not yet been examined, approved, and uploaded in the official depository of Ph.D. theses from Romanian universities.
